# Clustering of diet- and activity-related parenting practices: cross-sectional findings of the INPACT study

**DOI:** 10.1186/1479-5868-10-36

**Published:** 2013-03-25

**Authors:** Gerda Rodenburg, Anke Oenema, Stef PJ Kremers, Dike van de Mheen

**Affiliations:** 1 IVO Addiction Research Institute, Heemraadssingel 194, Rotterdam, 3021 DM, The Netherlands; 2 Erasmus Medical Center, Rotterdam, The Netherlands; 3 Department of Health Promotion, Maastricht University, Maastricht, The Netherlands

**Keywords:** Parenting practices, Clustering, Children, Dietary behaviour, Activity behaviour

## Abstract

**Background:**

Various diet- and activity-related parenting practices are positive determinants of child dietary and activity behaviour, including home availability, parental modelling and parental policies. There is evidence that parenting practices cluster *within* the dietary domain and *within* the activity domain. This study explores whether diet- and activity-related parenting practices cluster *across* the dietary and activity domain. Also examined is whether the clusters are related to child and parental background characteristics. Finally, to indicate the relevance of the clusters in influencing child dietary and activity behaviour, we examined whether clusters of parenting practices are related to these behaviours.

**Methods:**

Data were used from 1480 parent–child dyads participating in the Dutch IVO Nutrition and Physical Activity Child cohorT (INPACT). Parents of children aged 8–11 years completed questionnaires at home assessing their diet- and activity-related parenting practices, child and parental background characteristics, and child dietary and activity behaviours. Principal component analysis (PCA) was used to identify clusters of parenting practices. Backward regression analysis was used to examine the relationship between child and parental background characteristics with cluster scores, and partial correlations to examine associations between cluster scores and child dietary and activity behaviours.

**Results:**

PCA revealed five clusters of parenting practices: 1) high visibility and accessibility of screens and unhealthy food, 2) diet- and activity-related rules, 3) low availability of unhealthy food, 4) diet- and activity-related positive modelling, and 5) positive modelling on sports and fruit. Low parental education was associated with unhealthy cluster 1, while high(er) education was associated with healthy clusters 2, 3 and 5. Separate clusters were related to both child dietary and activity behaviour in the hypothesized directions: healthy clusters were positively related to obesity-reducing behaviours and negatively to obesity-inducing behaviours.

**Conclusion:**

Parenting practices cluster *across* the dietary and activity domain. Parental education can be seen as an indicator of a broader parental context in which clusters of parenting practices operate. Separate clusters are related to both child dietary and activity behaviour. Interventions that focus on clusters of parenting practices to assist parents (especially low-educated parents) in changing their child’s dietary and activity behaviour seems justified.

## Background

Diets rich in fruit and vegetables and an active lifestyle are associated with important health protective effects, including protection against some types of cancer, cardiovascular diseases, type 2 diabetes and overweight [1,2]. There is considerable evidence that children consume less fruit and vegetables than is recommended [3-7] and that they do not meet physical activity (PA) recommendations [8]. Because diet- and activity-related habits established in childhood often track through to adulthood [9-11], these energy balance-related behaviours (EBRBs) should be improved at an early age. Improvement of these behaviours requires understanding of the factors determining children’s EBRBs.

The home environment is a critical context for the development of children’s eating and activity behaviours [12-14]. Parents play a key role in shaping the home environment. In review studies on parental correlates of child fruit and vegetable consumption, the most consistently supported positive determinants of child and adolescent intake are parental dietary intake, parental modelling, home availability and accessibility, family rules, parental encouragement and parental education [12,15-18]. In addition, parental fat intake is a consistent and positive correlate of child fat intake [15]. Important positive parental correlates for child and adolescent PA are parental support, parental encouragement, paternal PA, maternal education level and family income [12,19,20]. Conceptually, such parental correlates can be divided into parenting practices (i.e. content-specific acts of parenting [21], such as rules about dietary intake or activity behaviour) and more general or distal parental factors (e.g., parental education and family income). The latter can be conceptualised as potential background variables or higher-order moderators of the relationship between parenting practices and child behaviour [7]. The current study focuses on clustering of parenting practices in relation to more distal parental factors.

There is some evidence that parenting practices co-occur or ‘cluster’. Gubbels et al. [22] found evidence for clustering of diet-related restrictive parenting practices, namely a cluster characterised by prohibition of the intake of various snacks and soft drinks, and a separate cluster characterised by prohibition of cookies and cake. A study by Gattshall et al. [23] showed interdependencies between diet-related parenting practices for fruit and vegetables, and between PA-related parenting practices, i.e. availability, accessibility, parental role modelling and parental policies. However, they did not study interdependencies between diet- and activity-related parenting practices. To our knowledge, no studies have used a clustering approach to examine both diet- and activity-related parenting practices, while studies on this topic are needed to elucidate whether parenting practices cluster across the dietary and activity domain (e.g. parental rule setting regarding snacks and screen time). Clustering across domains could point to a broader parental context in which the clusters of parenting practices operate, e.g. a parental context of health beliefs. The potential synergy between parenting practices that occur in clusters could result in more efficient interventions aimed at improving diet-and activity-related parenting practices, by applying an integrated approach that addresses multiple parenting practices simultaneously [24].

To elucidate how clusters of parenting practices may arise, it is important to examine factors related to the potential clusters of parenting practices. These factors can be both child- and parent-related. In previous studies, child gender [25,26], weight [26-30], food neophobia [31] and eating style (hungry or picky) [26], as well as parental body mass index (BMI), eduation level, parenting style, employment, ethnicity and parental age [22,26,27,31-37] were related to diet-related parenting practices, while child gender and activity style (active or not) [26], parental education level and working hours per week were related to activity-related parenting practices [26]. To test the magnitude of their relevance, it is also important to relate potential clusters of parenting practices to child dietary and activity behaviours. We chose to relate them to obesity-reducing, i.e. child fruit intake, child active commuting to school, child outdoor playing and child sports participation, as well as obesity-inducing behaviours, i.e. child snack and sugar-sweetenend beverage (SSB) intake, and child screen time [38].

The aim of this study was to examine clustering of parenting practices across the dietary and activity domain in parents of children aged 8–11 years. Children and their parents were recruited from rural and urban general primary schools in southern Netherlands. Apart from clustering of parenting practices, we examined whether these potential clusters are associated with child- and parent-related factors, and with child dietary and activity behaviours. Based on earlier studies we included child gender, age, ethnicity and weight, and parental BMI, education level and parenting style as factors that could potentially be associated with the clusters. We hypothesised that the parenting practices would cluster *within* and *across* the dietary and activity domain, and that healthy clusters would positively relate to obesity-reducing behaviours and negatively to obesity-inducing behaviours.

## Methods

### Study design, setting, participants and procedure

Data for this study were retrieved from the IVO Nutrition and Physical Activity Child cohorT (INPACT), for which approval was obtained from the Ethical Committee of the Erasmus MC (University Medical Center Rotterdam). INPACT is an observational study (initiated in 2008) focusing on modifiable determinants of overweight in the home environment of children aged 8–12 years in the Netherlands. INPACT was conducted among primary school children in southern Netherlands (Eindhoven area). In recruiting the schools in 2008, we collaborated with the Municipal Health Authority for Eindhoven and surrounding area (GGD Brabant-Zuidoost). The Municipal Health Authority invited all general primary schools in their service area to participate in the INPACT study. Of the 265 schools invited, 91 took part; the response rate from rural and urban schools was equal. The primary caregivers of third-grade students (aged ± 8 years) were invited to participate in the cohort study, together with their child. Of the 2948 parent–child dyads invited, 1839 (62.4%) gave written informed consent to participate in the INPACT study for four years. The study included four assessments, each separated by a one-year time interval, and started in the autumn of 2008 (baseline). In the assessments, primary caregivers completed a questionnaire at home, children completed a questionnaire at school, and qualified research assistants measured the children’s height and weight at school.

The present study was based on data from 2008 (baseline) and 2009 (second assessment). Parents reported on child and parental background characteristics (2008), on their energy balance-related parenting practices (partly in 2008 and partly in 2009) and on their children’s energy balance-related behaviours (2009). In addition, child BMI z-scores from 2008 were used, which were based on measured height and weight. Parent–child dyads with complete data from baseline to 2009 were included in the present study, resulting in 1480 parent–child dyads (80% of the original cohort). Logistic regression analyses on selective dropout from baseline to 2009 showed that parent–child dyads who were not native Dutch dropped out more often. There was no selective dropout regarding child age/gender and parental education level.

### Sample characteristics

At baseline (n=1839), 7% of the children were underweight, 79% had a normal weight and 14% were overweight, of which 3% were obese. The prevalence of overweight and obesity was similar to Dutch prevalence rates among primary school children [39]. The age of the children was 8 (77%) or 9 (20%) years (range 7–10 years, mean=8.2 years, SD=0.5). Boys (50.5%) and girls (49.5%) were represented in almost equal numbers. Of all children, 17% were from a non-Dutch ethnic background with one or both parents born abroad, of which 9% from non-western countries and 8% from western countries. Primary caregivers were predominantly female (92%). Of all primary caregivers, 21% had finished education at a low level, 45% at a medium level, 32% at a high level, and 2% at a non-specified level. Of the primary caregivers, 1% was underweight, 66% had a normal weight and 33% were overweight, of which 9% were obese.

### Measures

#### Diet- and activity-related parenting practices

Diet- and activity-related parenting practices were assessed with questionnaire items derived from the Dutch translation of the validated Home Environment Survey (HES) [23]. The home environment can be divided into a physical, socio-cultural, political and economic environment [40]; the HES assesses all of these, except for the economic home enviroment. The physical environment includes availability and accessibility of fruit, vegetables, snacks, SSBs and PA equipment (bicycle, roller skates, ball, etc.), the policital environment includes a scale for healthy eating parental policies (e.g. eating breakfast together with a child) and PA parental policies (e.g. encouraging a child to be physically active), and the socio-cultural environment includes a scale for healthy eating parental role modelling and PA parental role modelling. As suggested by Gattshall et al. [23] , we included a separate scale for parental role modelling of sedentary behaviour. In addition to Gattshall’s items on accessibility of PA equipment we included items on accessibility of sedentary equipment (television and computer). Moreover, we divided all accessibility measures into visibility (‘could be seen’) and accessibility (‘could easily be reached’) (Table 1), as visibility can function as a cue to action (Health Belief Model [41]), and thus be an important factor for influencing behaviour.

**Table 1:** Descriptives and scale information of key study variables (n=1839 for 2008 and n=1547 for 2009)

**Concept**	**Measurement year**	**Questions**	**Answering scale**	**Cronbach’s α**	**Median score (25th-75th perc.) / % yes**	**Range of scores**
		**(reference period: in the past 30 days)**				
***Diet-related parenting practices: physical home environment***						
Fruit availability	2008	How often do you have fruits available at home?	never (1) to always (5)	-	5.0 (5.0-5.0)	1.0-5.0
Fruit visibility	2008	Do you store fruits at home in a place where your child can easily see them, e.g. in a fruit bowl	never (1) to always (5)	-	5.0 (4.0-5.0)	1.0-5.0
Fruit accessibility	2008	Do you store fruits at home in a place that is easily accessible for you child?	never (1) to always (5)	-	5.0 (5.0-5.0)	1.0-5.0
Snack availability	2008	How often do you have sweet and savoury snacks available at home?^a^	never (1) to always (5)		4.5 (4.0-5.0)	1.0-5.0
Snack visibility	2008	Do you store sweet and savoury snacks at home in a place where your child can easily see them?^a^	never (1) to always (5)		2.0 (1.5-3.0)	1.0-5.0
Snack accessibility	2008	Do you store sweet and savoury snacks at home in a place that is easily accessible for your child?^a^	never (1) to always (5)	-	3.5 (2.5-5.0)	1.0-5.0
SSB availability	2008	How often do you have SSBs available at home?	never (1) to always (5)	-	5.0 (4.0-5.0)	1.0-5.0
SSB visibility	2008	Do you store SSBs at home in a place where your child can easily see them?	never (1) to always (5)	-	3.0 (2.0-5.0)	1.0-5.0
SSB accessibility	2008	Do you store SSBs at home in a place that is easily accessible for your child?	never (1) to always (5)	-	4.0 (3.0-5.0)	1.0-5.0
***Diet-related parenting practices: political home environment***						
Fruit rules	2008	Do you have a rule at home that your child should eat, in principle, 2 pieces of fruit per day?	no (0) or yes (1)	-	24.5	-
Snack rules	2008	Do you have a rule at home about how much and when your child is allowed to snack?^b^	no (0) or yes (1)	-	69.5^c^ 15.2^d^	-
SSB rules	2008	Do you have a rule at home about how much and when your child is allowed to drink SSBs?^b^	no (0) or yes (1)	-	58.6^c^ 15.5^d^	-
Healthy eating policies	2008	7 items, e.g., ‘How often do you eat breakfast with your child?’	never (1) to always (5)	0.60	4.1 (3.7-4.4)	1.1-5.0
***Diet-related parenting practices: socio-cultural home environment***						
Parental fruit intake	2008	Based on Food Frequency Questionnaires^e^	^e^	-	6.0 (3.3-10.5)	0-28 pieces
Parental snack intake	2008	Based on Food Frequency Questionnaires	^e^	-	6.0 (3.0-9.0)	0-35 pieces
Parental SSB intake	2008	Based on Food Frequency Questionnaires	^e^	-	1.0 (0.0-6.0)	0-42 glasses
Healthy eating role modelling	2009	12 items, e.g. ‘How often do you eat healthy meals or snacks while your child is around?	never (1) to always (5)	0.70	4.0 (3.8-4.2)	2.4-5.0
***Activity-related parenting practices: physical home environment***						
Availability of PA equipment and play spaces	2008	Which of the following toys/equipment does your child have? (list of 15 items, including skateboard, bicycle, skipping rope and outside play area)	no (0) or yes (1)		9.0 (7.0-10.0)	2-15
PA equipment visibility	2008	Do you store your child’s active toys out of sight when he/she is not using them? (reversed item)	never (1) to always (5)		4.0 (3.0-5.0)	1-5
PA equipment accessibility	2008	2 items, e.g., Do you store your child’s active toys in a place that is easily accessible for your child? (child needs no help getting them out)	never (1) to always (5)	0.72	5.0 (5.0-5.0)	1-5
Screen equipment availability in bedroom	2008	2 items: Does your child have a television/computer in his bedroom?	no (0) or yes (1)		7.5^c^	
					21.2^d^	
Screen equipment visibility	2008	2 items, e.g., Do you store your computer out of sight when it is not used? (reversed item)	never (1) to always (5)		4.5 (3.0-5.0)	1-5
Screen equipment accessibility	2008	2 items, e.g., Is the television mostly turned on at your place?	never (1) to always (5)		3.5 (3.0-4.0)	1-5
***Activity-related parenting practices: political home environment***						
Active transport rules	2008	Do you have a rule at home that your child, in principle, should go to school on foot or by bicycle?	no (0) or yes (1)		76.4	
Sports rules	2008	Do you have a rule at home that your child, in principle, should sport/be physically active?	no (0) or yes (1)		82.1	
Screen time rules	2008	4 items, e.g. Do you have a rule at home about how much your child is allowed to watch television?	no (0) or yes (1)		0.8 (0.3-1.0)	0-1
Physical activity policies	2008	5 items, e.g. How often do you verbally encourage your child to be physically active?	never (1) to always (5)	0.57	3.8 (3.4-4.2)	1.6-5.0
***Activity-related parenting practices: socio-cultural home environment***						
Parental active commuting days	2009	Two questions based on SQUASH s72], one about number of days per week walking to work and one about number of days per week cycling to work	number of days per week for each question (open questions)	-	0.0 (0.0-3.0)	0-7 days
Parental sports days	2009	Based on SQUASH; parents could indicate 4 types of sports they performed,	number of days per week for each sport indicated (open questions)	-	1.0 (0.0-2.0)	0-14 days
Parental PA apart from active commuting and sports	2009	Six questions based on SQUASH; number of days of walking, cycling, gardening and doing small jobs during leisure time per week, and number of days of physically heavy work and physically heavy housework per week	number of days per week for each question (open questions)	-	8.0 (5.0-12.0)	0-28 days
Parental screen days	2008	Two questions based on SQUASH, one about number of days per week watching television and one about number of days per week using the computer	number of days per week for each question (open questions)		10.0 (8.0-13.0)	0-14 days
Physical activity role modelling	2009	6 items, e.g. How often does your child see you being physically active (e.g. walking, cycling, playing sports)?	never (1) to always (5)	0.52	3.5 (3.3-3.7)	1.7-4.8
Sedentary behaviour role modelling	2009	2 items, e.g. How often does your child see you watching television?	never (1) to always (5)	0.48	3.0 (3.0-3.5)	1.0-5.0

*PA*: physical activity *SSB*: sugar-sweetened beverage.

^a^Separate questions for sweet snacks and for savoury snacks.

^b^Separate questions for ‘how much’ and for ‘when’.

^c^% ‘yes’ on both questions.

^d^% ‘yes’ on one of the two questions.

^e^Parental fruit, snack and SSB intake were assessed in the same way as child fruit, snack and SSB intake (see Methods section).

The HES assesses the physical environment for specific foods and PA equipment, while the political and socio-cultural environment are measured in a generic way (e.g. healthy eating policies/role modelling). In order to include specific measures, we also assessed parental rules for child dietary and activity behaviours as part of the political environment, and parental dietary and activity behaviours (role modelling) as part of the socio-cultural environment. These were assessed with questionnaire items derived from the Endorse study [42] (Table 1).

For all parenting practice measures, a higher score implied more policies/rules, role modelling, availability, etc. Table 1 presents additional information on measurement year, number of items, example items, response options, Cronbach’s alphas, and the means and standard deviations (SDs) of the various parenting practices assessed.

#### Child dietary and activity behaviours

Child fruit, snack and SSB intake in 2009 were assessed using several items from a validated Food Frequency Questionnaire (FFQ) designed to accurately assess energy intake of Dutch children aged 2–12 [43,44]. The validation study showed a correlation coefficient between the original questionnaire and the doubly labeled water method of 0.62. The way in which child fruit intake is assessed in this FFQ corresponds with earlier validated FFQs for fruit and vegetable intake [45,46]. The primary caregivers reported how many days in a normal week their children consumed 1) fruit (fresh, bottled and/or canned; no juice), 2) savoury snacks (e.g. potato crisps, peanuts and sausage rolls) in between meals, 3) sweet snacks (e.g. candies, chocolates and candy bars) in between meals, 4) cake or large biscuits in between meals, and 5) SSBs. Answering categories ranged from ‘none or less than 1 day a week’ to ‘7 days a week’. Additionally, they reported the number of servings consumed by their children on such a day. For fruit, answering categories ranged from ‘0 pieces per day’ to ‘more than 3 pieces per day’, by increments of half a piece of fruit. Reported consumption of more than 3 pieces per day (n=12) was recoded as 4 pieces. For savoury snacks, sweet snacks and cake or large biscuits, answering categories ranged from 0 to 10 servings a day. For SSBs, answering categories ranged from ‘0 glasses per day’ to ‘more than 5 glasses per day’, by increments of half a glass. It was specified that one glass equals 200 ml; one can equals 330 ml or 1.5 glasses; one bottle equals 500 ml or 2.5 glasses. Reported consumption of more than 5 glasses per day (n=7) was truncated to 6 pieces. Total child fruit and SSB intake were expressed in servings per week and calculated by multiplying frequency and quantity. Total child snack intake was also expressed in pieces per week and calculated by multiplying frequencies of savoury snacks, sweet snacks and cakes with their corresponding quantities, and summing these scores. Missing values on child fruit, snack and SSB intake were not imputed, because of the low number of missing values (1.0% at the highest, for child snacking).

Children’s activity behaviours were also reported by the primary caregiver, and based on a standard questionnaire for assessing children’s activity behaviour used in Dutch Youth Health Care [47]. Parents reported on how many days in a normal week their children 1) went to school on foot or by bicycle (active transport to school), 2) played outside, and 3) participated in a sport at a sports club. Children’s sedentary screen-time behaviour was assessed in a similar way with separate questions for watching television (including videos and DVDs) and playing on the computer. Total child screen time was calculated by summing television days and computer days, ranging from 0 to 14 days (e.g., if parents reported their child to watch television for 7 days per week and to play on the computer for 5 days per week, the child scored 12).

#### Child and parental background characteristics

Data on demographics were primarily collected in the parent questionnaire of 2008. Child age was measured in years by subtracting the date of questionnaire completion from the child’s birth date. To assess the child’s ethnic background, the primary caregiver reported the country of origin of both parents. According to standard procedures of Statistics Netherlands [48], a child was classified as native Dutch if both parents were born in the Netherlands, as a western immigrant if at least one parent was born outside the Netherlands but inside Europe, and as a non-western immigrant if at least one parent was born in Turkey, Africa, Latin America or Asia. The primary caregiver also reported on his/her highest level of education. According to international classification systems [49], parental education level was defined as low (primary school and lower vocational/lower general secondary education), medium (intermediate vocational education, higher general secondary education and university prep), high (higher vocational education and university), or non-defined. To assess parental BMI, the primary caregiver reported his/her own height and weight. He/she also reported whether he/she was the child’s biological parent. Parental BMI (for biological parents only) was calculated on the basis of these answers.

Parenting style was measured using the Dutch translation [50] of an instrument based on earlier work by Steinberg et al. [51,52], which is used in many studies worldwide [50,53-55]. This 22-item measure assessed three parenting-style dimensions: support (e.g. ‘When my child gets a low grade in school, I offer to help him/her’), behavioural control (e.g. ‘I know exactly what my child does in his/her free time’ and psychological control (e.g. ‘I make my child feel guilty when he/she gets a low grade in school’) (see [56] for additional information on the parenting style instrument used).

In addition to questionnaire data, child BMI was based on the child’s height and weight: i.e. weight (kg)/height (m)^2^, as measured by the qualified research assistants in 2008. Children were measured at school according to standard procedures in light clothing without shoes, to the nearest 0.1 kg and 0.1 cm. Weight was measured with an electronic flat scale (Seca 840; Beenhakker, Rotterdam, the Netherlands) and height with a mobile measuring ruler (Seca 214; Beenhakker, Rotterdam, the Netherlands). BMI z-scores were calculated [57] based on age and gender-specific values from the 1997 National Growth Study in the Netherlands [58].

### Strategy for analyses

All analyses were performed using IBM SPSS Statistics version 19.0. Cases with missing values were excluded per analysis. To describe the study population, we computed medians, interquartile ranges and percentages for socio-demographic variables and child dietary and activity behaviours.

Principal component analysis (PCA) with oblique rotation was performed to examine clustering of diet-related and activity-related parenting practises. Oblique rotation was chosen because of the expected association between the extracted components [59]. A scree plot was used to determine the number of components. Items with absolute component loadings larger than 0.4 were considered part of the component, in line with previous research [59]. Cluster scores were computed for each child as each parenting practice measure multiplied by their corresponding component loading [60]. The parenting practice cluster scores were then used as separate dependent variables in backward linear regression analyses, to examine the relationship with parental characteristics (parental education level, parental BMI at baseline and parenting style dimensions) and child characteristics (gender, age, ethnicity and BMI z-score at baseline). Partial correlation was used to assess the associations between cluster scores and child dietary and activity behaviours. These analyses were corrected for the child and parent background characteristics mentioned above.

## Results

Children had an average weekly fruit consumption of 7.4 pieces (SD=4.2; range: 0–28), an average weekly snack intake of 9.7 pieces (SD=5.8; range: 0–35) and an average weekly SSB intake of 9.1 glasses (SD=8.3; range: 0–42). Only 15% of the children met the recommended Dutch norm of at least 14 pieces of fruit per week [61]. On average, children went to school on foot or by bicycle on 4.3 days per week (SD=1.3; range: 0–5), played outside on 6.6 days per week (SD=0.8, range: 0–7), participated in a sport at a sports club on 2.5 days per week (SD=1.3; range: 0–7), watched television on 6.7 days per week (SD=0.89, range: 0–7) and played on the computer on 4.7 days per week (SD=2.0; range 0–7). Of all children, 75% commuted to school in an active way all 5 days of the school week, 77% played outside all 7 days of the week, 86% watched television all 7 days of the week and 32% played on the computer all 7 days of the week.

PCA revealed 5 parenting practice clusters (Table 2). The first cluster included a high *visibility* and *accessibility* of SSB and snacks, a high availability of screens in the child’s bedroom and a low score on parental healthy eating policies (‘high visibility and accessibility of screens and unhealthy food’ cluster). The second cluster included snack and SSB *rules*, screen-time rules and sports rules (‘diet- and activity-related rules’ cluster). The third cluster combined a low *availability* of snacks and SSBs with a low accessibility of snacks and SSBs (‘low availability of unhealthy food’ cluster). The fourth cluster included parental *modelling* of healthy eating, as well as low parental sedentary modelling, low parental snack intake and high accessibility of PA equipment (‘diet- and activity-related positive modelling’ cluster). The final cluster combined high parental sports days and high parental fruit intake with positive PA *modelling* (‘positive modelling on sports and fruit’ cluster). The five parenting practice clusters explained 32.0% of the variance in the original items. Cluster 1 and 2 were negatively correlated (r=-0.16), while cluster 2 and 4 (r=0.17) and 4 and 5 (r=0.12) were positively correlated. The remaining combinations of clusters were not related (r<0.10).

**Table 2:** Component loadings of principal component analysis on diet- and activity-related parenting practices (n=1059, missings list wise)

**Parenting practices**	**Cluster 1: High visibility and accessibility of screens and unhealthy food**	**Cluster 2: Diet- and activity- related rules**	**Cluster 3: Low availability of unhealthy food**	**Cluster 4: Diet- and activity-related positive modelling**	**Cluster 5: Positive modelling on sports and fruit**
SSB visibility	**0.768**	0.100	-0.091	0.050	0.028
Snack visibility	**0.736**	0.116	-0.145	0.026	0.023
Healthy eating policies	**-0.496**	0.137	-0.262	0.160	0.077
Screen equipment availability in bedroom	**0.440**	-0.068	0.075	0.046	-0.148
Screen equipment accessibility	0.329	-0.180	-0.128	-0.224	0.059
Availability of PA equipment and play spaces	-0.320	0.033	-0.213	0.000	0.249
Parental SSB intake	0.250	-0.039	-0.222	-0.148	-0.191
Snack rules	0.049	**0.753**	-0.099	-0.117	-0.030
SSB rules	-0.007	**0.734**	-0.057	-0.016	-0.038
Screen time rules	-0.036	**0.698**	0.068	-0.083	-0.006
Sports rules	-0.111	**0.426**	-0.126	0.016	0.167
Active transport rules	0.040	0.347	-0.215	0.193	-0.013
PA policies	0.140	0.297	0.132	0.084	0.272
Parental active commuting days	0.022	0.187	0.086	-0.022	-0.028
Snack availability	-0.086	0.000	**-0.674**	-0.022	-0.005
SSB availability	0.079	-0.036	**-0.649**	0.021	-0.106
SSB accessibility	**0.478**	-0.043	**-0.513**	0.038	-0.016
Snack accessibility	**0.424**	0.032	**-0.510**	-0.053	0.044
Healthy eating role modelling	-0.080	-0.015	-0.003	**0.604**	0.030
Sedentary behaviour role modelling	0.090	-0.118	-0.068	**-0.564**	0.287
Parental snack intake	-0.078	0.097	-0.349	**-0.462**	-0.110
PA equipment accessibility	0.004	-0.046	-0.243	**0.403**	0.218
PA equipment visibility	0.063	-0.067	-0.038	0.384	0.021
Parental PA days apart from active commuting and sports	0.012	-0.023	-0.031	0.373	0.065
Parental screen days	0.028	-0.107	-0.224	-0.361	0.108
Screen equipment visibility	0.168	-0.117	0.045	-0.226	0.216
Parental sports days	-0.116	0.010	0.094	-0.207	**0.547**
PA role modelling	-0.081	0.002	0.002	0.193	**0.541**
Parental fruit intake	-0.079	0.129	0.195	0.027	**0.445**
Fruit availability	-0.166	0.039	-0.212	0.011	0.358
Fruit accessibility	0.079	-0.165	-0.136	0.139	0.330
Fruit rules	0.118	0.268	0.178	0.154	0.316
Fruit visibility	0.274	0.010	0.253	0.002	0.283

PA: physical activity SSB: sugar-sweetened beverage.

Data printed **bold** indicate absolute component loadings larger than 0.4 (= part of the component).

Variance explained by component 1 = 10.6%; variance explained by component 2 = 6.4%; variance explained by component 3 = 5.7%; variance explained by component 4 = 5.0% and variance explained by component 5 = 4.3%.

Results of the regression analyses with the cluster scores as dependent variables (Table 3) showed that parents of non-western and western immigrant children, parents with a higher BMI, lower education and parents who used higher levels of psychological control scored significantly higher on the ‘high visibility and accessibility of screens and unhealthy food’ cluster. The ‘diet- and activity-related rules’ cluster was positively associated with a high parental education and with higher levels of behavioural control. Parents of non-western and western immigrant children as well as high-educated parents scored significantly higher on the ‘low availability of unhealthy food’ cluster. The ‘diet- and activity-related positive modelling’ cluster was positively associated with child BMI z-scores, negatively with parental BMI and psychological control, and positively with behavioural control. Finally, middle and high-educated parents and parents who used higher levels of behavioural control scored significantly higher on sports- and fruit-related positive modelling (cluster 5).

**Table 3:** **Child and parental characteristics related to cluster scores (standardized regression coefficients backward regression), n=981**^**1**^

	**Cluster 1: Hig visibility and accessibility of screens and unhealthy food**^**2**^	**Cluster 2: Diet- and activity- related rules**^**3**^	**Cluster 3: Low availability of unhealthy food**^**4**^	**Cluster 4: Diet- and activity- related positive modelling**^**5**^	**Cluster 5: Positive modelling on sports and fruit**^**6**^
***Child characteristics:***					
Ethnicity: non-western (1) vs native Dutch (0)	0.20***	-	0.23***	-	-
Ethnicity: western (1) vs native Dutch (0)	0.10**	-	0.14***	-	-
Child BMI z-score at baseline (2008)	-	-	-	0.08*	-
***Parental background characteristics:***					
Parental BMI	0.12***	-	-	-0.10**	-
Education: middle (1) vs low (0)	-0.17***	-	-	-	0.10*
Education: high (1) vs low (0)	-0.25***	0.15***	0.15***	-	0.20***
***Parenting style dimensions:***					
Psychological control	0.11***	-	-	-0.12***	-
Behavioural control	-	0.14***	-	0.18***	0.10**

^1^child characteristics: gender, age, ethnicity, BMI z-score; parental characteristics: parental education level, parental BMI; parenting style dimensions;

* correlation is significant at the 0.05 level (2-tailed); ** correlation is significant at the 0.01 level (2-tailed); *** correlation is significant at the 0.001 level (2-tailed).

^2^R^2^=0.14.

^3^R^2^=0.03.

^4^R^2^=0.09.

^5^R^2^=0.07.

^6^R^2^=0.04.

As shown in Table 4, partial correlations revealed that the cluster high in visibility and accessibility of screens and unhealthy food was negatively associated with child fruit intake, and positively with child snack intake, SSB intake and screen time. The diet- and activity-related rules cluster was positively associated with child fruit intake and child active transport, but negatively associated with child snack and SSB intake and child screen time. The cluster of low availability of unhealthy food showed negative associations with child snack and SSB intake as well as with child active transport and screen time. Positive parental modelling on dietary, PA and sedentary behaviour (cluster 4) showed positive associations with child fruit intake, child active transport and child outdoor playing, and negative associations with child snack and SSB intake and with child screen time. Positive parental modelling on sports and fruit was positively associated with child fruit intake and sports, as well as with child outdoor playing, and negatively with child SSB intake.

**Table 4:** Associations between clusters of diet and activity-related parenting practices and child dietary and activity behaviours (partial correlation coefficients), n=10131

**Cluster**	**Child fruit intake**	**Child snack intake**	**Child SSB intake**	**Child active transport to school**	**Child outdoor playing**	**Child sports participation at a sports club**	**Child screen time**
1: High visibility and accessibility of screens and unhealthy food	-0.08*	0.07*	0.10**	-0.04	-0.01	0.01	0.11**
2: Diet- and activity- related rules	0.08**	-0.08*	-0.12***	0.15***	0.02	0.03	-0.11**
3: Low availability of unhealthy food	0.06	-0.19***	-0.11***	-0.09**	-0.01	-0.05	-0.11**
4: Diet- and activity- related positive modelling	0.11***	-0.26***	-0.15***	0.20***	0.14***	0.01	-0.19***
5: Positive modeling on sports and fruit	0.30***	-0.04	-0.07*	0.05	0.12***	0.16***	0.03

*SSB*: sugar-sweetened beverage.

^1^Adjusted for child characteristics (gender, age, ethnicity and BMI z-score at baseline) and parental characteristics (parental education level, parental BMI at baseline and parenting style dimensions). Child dietary and activity behaviours were assessed in 2009 (=second assessment).

## Discussion

This study investigated the clustering of parenting practices across the dietary and activity domain. We also examined whether these clusters are associated with child- and parent-related factors, and with child dietary and activity behaviours. As hypothesised, we found evidence for clustering *within* the dietary domain (e.g. clustering of SSB- and snack-related parenting practices) and *within* the activity domain (e.g. clustering of screen time rules and sports rules), which is in line with the few studies that reported on interdependencies between diet-related parenting practices [22,23] and between activity-related parenting practices [23]. A new finding is that parenting practices cluster *across* domains: four out of five clusters included both diet- and activity-related parenting practices. In addition, parenting practices cluster on the type of home enviroment: two clusters represented the physical home environment (‘high visibility and accessibility of screens and unhealthy food’ and ‘low availability of unhealthy food’), one represented the policital home environment (the ‘diet- and activity-related rules’ cluster) and the two parental modelling clusters represented the socio-cultural home environment. These new findings are very relevant in terms of broadening the scientific knowledge base on the topic of parenting practices.

In the present study, parental modelling was assessed in two ways: using role modelling scales of the HES [23] and parent’s own behaviour. The diet- and activity-related positive modelling cluster (cluster 4) included two parental role modelling scales. They referred to parental healthy eating and sedentary behaviour *that was directly observed by the child*[23] (see example items in Table 1). This might imply the assessment of a more conscious way of parenting (a parenting practice) than when parental modelling is assessed by a parent’s own behaviour.

The diet- and activity-related positive modelling cluster (cluster 4) was more likely to be found in parents of heavier children who are lighter themselves, and express more behavioural control and less psychological control. This suggests that this might be a parental strategy in response to their child’s higher weight, particularly in normal weight parents. Similarly, diet- and activity-related positive modelling may be a stable parental strategy, reflecting normal weight parents’ own way of living [62], based on health beliefs. Finally, it may not be a parental strategy aimed at healthy dietary and activity behaviour in children, but rather a more unconscious way of parenting based on, for example, habits formed in early life. Similarly, the ‘diet- and activity-related rules’ cluster (cluster 2) might be a parental strategy based on health beliefs, but rule setting in the dietary and activity domain could also be part of a broader parental context of rule setting, based on, for example, parenting beliefs of strictness and involvement. This is supported by the finding that cluster 2 was positively related to behavioural control, which is an indicator of parental involvement.

There is evidence that parental education level indicates a broader parental context in which parenting practices operate [7,63]. A non-supportive parental context might be reflected in cluster 1, the unhealthy cluster of making screens and unhealthy food visible and accessible at home, which was more likely to be found in low-educated parents, but also in minority groups, parents with a higher BMI and parents who use more psychological control (all found to be associated with a higher child weight and/or unhealthy lifestyle (e.g., [15,56,64]). In contrast, healthy clusters are generally more likely to be found in high(er)-educated parents. These findings are consistent with the well-established relationship between socioeconomic position and health, stating that the socioeconomically better-off do better on most measures of health status [65]. Our findings also suggest that low-educated parents are an important target group for intervention development aimed at improving clustered parenting practices. However, because of the explorative nature of our study, the results cannot yet be translated into far reaching implications for public health. Before interventions can be developed, more studies are needed to elucidate how clusters of parenting practices arise (e.g. whether execution of parenting practices is a deliberate or a more unconscious process, whether parents adapt their practices or not and based on which indicators) and how they can be influenced, especially in low-educated parents. Apart from individual factors (e.g. a lack of knowledge and skills about parenting or a lack of health consciousness), exploring the social context of low-educated parents may elucidate why they have less-favourable parenting practices than high-educated parents. Ways in which the social context of low-educated parents can place constraints on their individual choices is by shaping social norms and by providing less opportunities to engage in healthy behaviours. This may influence their own health behaviour [66], but also their health-related parenting practices. For example, group norms may ensure that low-educated parents pursue other values than health values, and because of neighbourhood safety problems, they may not encourage their children to play outside. To better understand parenting practices in low-educated parents, future studies should explore the influence of the social context.

To indicate the magnitude of their relevance, we examined whether the five clusters were related to child dietary and activity behaviour. We found that the separate clusters were related to both child dietary behaviour and child activity behaviour and, overall, in the hypothesised direction: the ‘high visibility and accessibility of screens and unhealthy food’ cluster was positively related to obesity-inducing behaviour (i.c. child snack intake, SSB intake and screen time) and negatively to obesity-reducing behaviour (i.c. child fruit intake), while the remaining healthy clusters were negatively related to obesity-inducing behaviour and positively to obesity-reducing behaviour. The strongest associations were found in the positive modelling clusters. Diet- and activity-related positive modelling was found to have the strongest associations with child snack intake, SSB intake, active transporting to school, outdoor playing and screen time, while positive modelling on sports and fruit was strongest related to child fruit intake and child sports participation. This underlines the potential of a clustered approach of parental modelling in the dietary and activity domain as a parental strategy to (subtly) improve children’s dietary and activity behaviour. However, in low-educated parents this implies changing their own behaviour, which may be harder to accomplish than, for example, introducing parental rules in the dietary and activity domain. As the diet- and activity-related rules cluster was positively related to cluster 4, setting rules might eventually be an indirect way to change parental role modelling in a positive way.

Our study has the strength of combining diet- and activity-related parenting practices, higher-order parental factors and child dietary and activity behaviours in one study, which is exceptional in this field of research[18]. In addition, our clustering approach, which is new in studies on parenting practices, seems to have potential as a starting point for interventions to assist parents in changing their child’s dietary and activity behaviour. Such interventions could be more efficient because of the synergic effect of a clustered approach. Nevertheless, some limitations should be mentioned. First, diet- and activity-related parenting practices were reported by the primary caregiver (mostly the mother), while research shows that, for example, for child PA paternal and not maternal role modelling is the main determinant [20]. Future studies should (ideally) include both parents to examine whether fathers and mothers have a differential influence on child dietary and activity behaviour. Second, there was low variability in responses for some parenting practices, e.g. fruit availability and accessibility, which might explain why these parenting practices are not part of a cluster. However, this could also be explained by analytical choices, namely choosing a cut-off point for component loadings of 0.4. Although this is in line with recommendations [67], cut-off points in previous studies ranged from 0.2 to 0.6 [68]. If, for example, a cut-off point of 0.3 had been used in our study, fruit availability, fruit accessibility as well as fruit rules would have been included in the positive modelling on sports and fruit cluster. Third, Cronbach’s alpha values of some of our parenting practices scales were relatively low. Although a Cronbach’s alpha ≥.6 is generally considered acceptable [69], some authors advocate different cut-off points. Finally, child dietary and activity behaviours were proxy reports of primary caregivers, which may evoke social desirability bias and lead to overestimation of obesity-reducing behaviours and underestemiantion of obesity-inducing behaviours [70-72]. In addition, child activity behaviours were reported in days per week which may not accurately reflect behaviour duration or energy expenditure, especially for outdoor playing and screen time.

## Conclusions

The current study shows that parenting practices cluster on the type of home environment (i.e. physical, political and socio-cultural) while cutting across the dietary and activity domain. Several parental characteristics were related to the separate clusters, of which parental education level could be seen as an indicator of a broader parental context in which the clusters of parenting practices operate. A low parental education level was associated with the only unhealthy cluster, while a high(er) education level was associated with healthy clusters. Separate clusters were related to both child dietary behaviour and child activity behaviour in the hypothesised directions, indicating the relevance of the clusters in influencing child behaviour. Interventions that focus on clusters of parenting practices to assist parents, especially low-educated parents, in changing their child’s dietary and activity behaviour seems justified, but more studies are needed to further elucidate how clusters arise and how they can be influenced.

## Abbreviations

BMI: Body mass index; EBRB: Energy balance-related behaviour; FFQ: Food frequency questionnaire; HES: Home environment survey; INPACT IVO: Nutrition and physical activity child cohort; PA: Physical activity; SSB: Sugar-sweetened beverage

## Competing interests

The authors declare that they have no competing interests.

## Authors’ contributions

GR, AO, SPJK and DvdM designed the study. GR conducted the INPACT study and performed the statistical analyses. GR, AO, SPJK and DvdM wrote the manuscript. All authors read and approved the final manuscript.

## References

[B1] World Health Organization Diet, Nutrition and the Prevention of Chronic Diseases 2003 Geneva: Report of a Joint WHO/FAO Expert Consultation

[B2] World Health Organization The World Health Report. Reducing Risks, Promoting Healthy Life 2002 Geneva: WHO

[B3] Currie C Roberts C Morgan A Smith R Settertobulte W Samdal O Rasmussen VB Young people’s health in context. Health Behaviour in School-aged Children (HBSC) study 2004 Copenhagen: International report from the 2001/2002 survey

[B4] Guenther P Dodd K Reedy J Krebssmith S Most Americans Eat Much Less than Recommended Amounts of Fruits and Vegetables J Am Diet Ass 2006 106 371 1379 10.1016/j.jada.2006.06.002 16963342

[B5] Huybrechts L Matthys C Vereecken CA Matthys L Temme E Van Oyen H Food intakes by pre-school children in Flanders compared with recommendations Int J Environ Res Public Health 2008 5 243 257 10.3390/ijerph5040243 19190355 PMC2672311

[B6] Jones LR Steer CD Rogers IS Emmett PM Influences on child fruit and vegetable intake: sociodemographic, parental and child factors in a longitudinal cohort study Public Health Nutr 2010 13 1122 1130 10.1017/S1368980010000133 20196909

[B7] Rodenburg G Oenema A Kremers SPJ Van de Mheen H Parental and child fruit consumption in the context of general parenting, parental education and ethnic background Appetite 2012 58 364 372 10.1016/j.appet.2011.11.001 22094182

[B8] Verloigne M Van Lippevelde W Maes L Yildirim M Chinapaw M Manios Y Androutsos O Kovacs E Bringolf-Isler B Brug J De Bourdeaudhuij I Levels of physical activity and sedentary time among 10- to 12-year-old boys and girls across 5 European countries using accelerometers: an observational study within the ENERGY-project Int J Behav Nutr Phys Act 2012 9 34 10.1186/1479-5868-9-34 22462550 PMC3359200

[B9] Mikkilä V Räsänen L Raitakari OT Pietinen P Viikari J Longitudinal changes in diet from childhood into adulthood with respect to risk of cardiovascular diseases: The Cardiovascular Risk in Young Finns Study Eur J Clin Nutr 2004 58 1038 1045 10.1038/sj.ejcn.1601929 15220946

[B10] Kelder SH Perry CL Klepp KI Lytle LL Longitudinal tracking of adolescent smoking, physical activity, and food choice behaviors Am J Public Health 1994 84 1121 1126 10.2105/AJPH.84.7.1121 8017536 PMC1614729

[B11] Patterson E Wärnberg J Kearney J Sjöström M The tracking of dietary intakes of children and adolescents in Sweden over six years: the European Youth Heart Study Int J Behav Nutr Phys Act 2009 6 91 10.1186/1479-5868-6-91 20003331 PMC2797763

[B12] Pinard CA Yaroch AL Hart MH Serrano EL McFerren MM Estabrooks PA Measures of the home environment related to childhood obesity: a systematic review Public Health Nutr 2011 15 95 107 10.1017/S1368980011002059 21899786

[B13] Kral TVE Rauh EM Eating behaviors of children in the context of their family environment Physiol Behav 2010 100 567 573 10.1016/j.physbeh.2010.04.031 20457172 PMC2896260

[B14] Golan M Crow S Parents Are Key Players in the Prevention and Treatment of Weight-related Problems Nutr Rev 2004 62 39 50 10.1111/j.1753-4887.2004.tb00005.x 14995056

[B15] Van der Horst K Oenema A Ferreira I Wendel-Vos W Giskes K Van Lenthe FJ Brug J A systematic review of environmental correlates of obesity-related dietary behaviors in youth Health Educ Res 2007 22 203 226 16861362 10.1093/her/cyl069

[B16] Rasmussen M Krolner R Klepp KI Lytle L Brug J Bere E Due P Determinants of fruit and vegetable consumption among children and adolescents: a review of the literature. Part I: quantitative studies Int J Behav Nutr Phys Act 2006 3 22 10.1186/1479-5868-3-22 16904006 PMC1564033

[B17] Pearson N Biddle SJH Gorely T Family correlates of fruit and vegetable consumption in children and adolescents: a systematic review Public Health Nutr 2008 12 267 283 18559129 10.1017/S1368980008002589

[B18] Ventura AK Birch LL Does parenting affect children’s eating and weight status? Int J Behav Nutr Phys Act 2008 5 15 10.1186/1479-5868-5-15 18346282 PMC2276506

[B19] Sallis JF A review of correlates of physical activity of children and adolescents Med Sci Sports Exerc 2000 32 963 975 10795788 10.1097/00005768-200005000-00014

[B20] Ferreira I Van der Horst K Environmental correlates of physical activity in youth–a review and update Obes Rev 2007 8 129 154 10.1111/j.1467-789X.2006.00264.x 17300279

[B21] Darling N Steinberg L Parenting style as context: An integrative model Psych Bull 1993 113 487 496

[B22] Gubbels JS Kremers SPJ Stafleu A Dagnelie PC Goldbohm RA De Vries NK Thijs C Diet-related restrictive parenting practices. Impact on dietary intake of 2-year-old children and interactions with child characteristics Appetite 2009 52 423 429 10.1016/j.appet.2008.12.002 19114065

[B23] Gattshall ML Shoup JA Marshall JA Crane LA Estabrooks PA Validation of a survey instrument to assess home environments for physical activity and healthy eating in overweight children Int J Behav Nutr Phys Act 2008 5 3 10.1186/1479-5868-5-3 18190709 PMC2253552

[B24] Kremers SPJ De Bruijn G-J Schaalma H Brug J Clustering of energy balance-related behaviours and their intrapersonal determinants Psychol Health 2004 19 595 606 10.1080/08870440412331279630

[B25] Tiggemann M Lowes J Predictors of maternal control over children’s eating behaviour Appetite 2002 39 1 7 10.1006/appe.2002.0487 12160560

[B26] Gubbels JS Kremers SPJ Stafleu A De Vries SI Goldbohm RA Dagnelie PC De Vries NK De Buuren S Thijs C Association between parenting practices and children’s dietary intake, activity behavior and development of body mass index: the KOALA Birth Cohort Study Int J Behav Nutr Phys Act 2011 8 18 10.1186/1479-5868-8-18 21401954 PMC3065396

[B27] Kröller K Warschburger P Maternal feeding strategies and child’s food intake: considering weight and demographic influences using structural equation modeling Int J Behav Nutr Phys Act 2009 6 78 10.1186/1479-5868-6-78 19930607 PMC2785754

[B28] Stang J Rehorst J Golicic M Parental feeding practices and risk of childhood overweight in girls: implications for dietetics practice J Am Diet Ass 2004 104 1076 1079 10.1016/j.jada.2004.03.030 15215764

[B29] Wardle J Carnell S Parental feeding practices and children’s weight Acta Paediatr 2007 96 454 5 11 10.1111/j.1651-2227.2007.00163.x 17313408

[B30] Brann L Skinner J More controlling child-feeding practices are found among parents of boys with an average body mass index compared with parents of boys with a high body mass J Am Diet Ass 2005 105 1411 1416 10.1016/j.jada.2005.06.005 16129082

[B31] Blissett J Relationships between parenting style, feeding style and feeding practices and fruit and vegetable consumption in early childhood Appetite 2011 57 826 831 10.1016/j.appet.2011.05.318 21651932

[B32] Arredondo E Elder J Is parenting style related to children’s healthy eating and physical activity in Latino families? Health Educ Res 2006 21 862 871 10.1093/her/cyl110 17032706

[B33] Hupkens C Knibbe R Class differences in the food rules mothers impose on their children: a cross-national study Soc Sc Med 1998 47 1331 1339 10.1016/S0277-9536(98)00211-1 9783876

[B34] Brown KA Ogden J Vögele C Gibson EL The role of parental control practices in explaining children’s diet and BMI Appetite 2008 50 252 259 10.1016/j.appet.2007.07.010 17804116

[B35] Liem D Mars M De Graaf C Sweet preferences and sugar consumption of 4-and 5-year-old children: role of parents Appetite 2004 43 235 245 10.1016/j.appet.2004.05.005 15527925

[B36] Montgomery C Jackson D Parental feeding style, energy intake and weight status in young Scottish children Br J Nutr 2006 96 1149 1153 10.1017/BJN20061968 17181891

[B37] Keller K Pietrobelli A Maternal restriction of children’s eating and encouragements to eat as the “non-shared environment”: a pilot study using the child feeding questionnaire Int J Obes 2006 30 1670 1675 10.1038/sj.ijo.0803318 16568136

[B38] Rennie KL Johnson L Jebb SA Behavioural determinants of obesity. Best practice & research Clin Endocr Metab 2005 19 343 358 10.1016/j.beem.2005.04.003 16150379

[B39] Brug J Van Stralen MM Chinapaw MJM De Bourdeaudhuij I Lien N Bere E Singh AS Maes L Moreno L Jan N Kovacs E Lobstein T Manios Y Te Velde SJ Differences in weight status and energy-balance related behaviours according to ethnic background among adolescents in seven countries in Europe: the ENERGY-project Pediatric Obes 2012 7 399 411 10.1111/j.2047-6310.2012.00067.x 22730265

[B40] Swinburn B Egger G Raza F Dissecting obesogenic environments: the development and application of a framework for identifying and prioritizing environmental interventions for obesity Prev Med 1999 29 563 570 10.1006/pmed.1999.0585 10600438

[B41] Glanz K Rimer B Lewis F Health Behavior and Health Education. Theory, Research and Practice 2002 San Fransisco: Wiley & Sons

[B42] Horst K Oenema A Looij-Jansen P Brug J The ENDORSE study: research into environmental determinants of obesity related behaviors in Rotterdam schoolchildren BMC Public Health 2008 8 142 10.1186/1471-2458-8-142 18442370 PMC2413223

[B43] Dutman AE Stafleu A Kruizinga A Brants HA Westerterp KR Kistemaker C Meuling WJ Goldbohm RA Validation of an FFQ and options for data processing using the doubly labelled water method in children Public Health Nutr 2011 14 410 417 10.1017/S1368980010002119 20707949

[B44] Brants H Stafleu A Ter Doest D Hulshof K Thijs C Ontwikkeling van een voedselfrequentievragenlijst: energie-inneming van kinderen van 2 tot en met 12 jaar (Development of a food frequency questionnaire: energy intake of children aged 2 to 12 years) Voeding Nu 2006 2 25 28

[B45] Bogers RP Van Assema P Kester AD Westerterp KR Dagnelie PC Reproducibility, validity, and responsiveness to change of a short questionnaire for measuring fruit and vegetable intake Am J Epidem 2004 159 900 909 10.1093/aje/kwh123 15105183

[B46] Haraldsdottir J Thorsdottir I De Almeida MDV Maes L Perez R Carmen P Elmadfa I Frost Andersen L Validity and Reproducibility of a Precoded Questionnaire to Assess Fruit and Vegetable Intake in European 11- to 12-Year-Old Schoolchildren Ann Nutr Metab 2005 49 221 227 10.1159/000087276 16088085

[B47] GGD Nederland, RIVM, Actiz Monitor Jeugdgezondheid: Standaard Vraagstelling Bewegen (Monitor Youth Health: Standard Questionnaire Physcial Activity) 2009 Utrecht: GGD Nederland

[B48] Netherlands S Standaarddefinitie allochtonen (Standard definition immigrants) Hoe doet het CBS dat nou? 2000 Voorburg: Statistics Netherlands 24 25

[B49] Eurostat Task Force on Core Social Variables. Final Report 2007 Luxembourg: European Communitees

[B50] Beyers W Goossens L Emotional autonomy, psychosocial adjustment and parenting: Interactions, moderating and mediating effects J Adolesc 1999 22 753 769 10.1006/jado.1999.0268 10579888

[B51] Steinberg L Elmen JD Mounts NS Authoritative parenting, psychosocial maturity, and academic success among adolescents. Child Dev 1989 60 1424 1436 10.2307/1130932 2612251

[B52] Lamborn SD Mounts NS Steinberg L Dornbusch SM Patterns of competence and adjustment among adolescents from authoritative, authoritarian, indulgent, and neglectful families Child Dev 1991 62 1049 1065 10.2307/1131151 1756655

[B53] Huver RME Engels RCME Vermulst AA De Vries H Is parenting style a context for smoking-specific parenting practices? Drug Alcohol Depen 2007 89 116 125 10.1016/j.drugalcdep.2006.12.005 17300879

[B54] Kremers SPJ Brug J De Vries H Engels RCM Parenting style and adolescent fruit consumption Appetite 2003 41 43 50 10.1016/S0195-6663(03)00038-2 12880620

[B55] Pearson N Atkin AJ Biddle SJ Gorely T Edwardson C Parenting styles, family structure and adolescent dietary behaviour Public Health Nutr 2010 13 1245 1253 10.1017/S1368980009992217 19954574

[B56] Rodenburg G Kremers SPJ Oenema A Van de Mheen H Psychological control by parents is associated with a higher child weight Int J Pediatr Ob 2011 6 442 449 10.3109/17477166.2011.590203 21780869

[B57] Cole TJ Bellizzi MC Flegal KM Dietz WH Establishing a standard definition for child overweight and obesity worldwide: international survey BMJ 2000 320 1240 1246 10.1136/bmj.320.7244.1240 10797032 PMC27365

[B58] Fredriks AM Van Buuren S Wit JM Verloove-Vanhorick SP Body index measurements in 1996–7 compared with 1980 Arch Dis Child 2000 82 107 112 10.1136/adc.82.2.107 10648362 PMC1718204

[B59] Field A Discovering Statistics Using SPSS 2005 2 London: SAGE Publications Ltd

[B60] Lioret S Touvier M Lafay L Volatier J-L Maire B Dietary and Physical Activity Patterns in French Children Are Related to Overweight and Socioeconomic Status J Nutr 2008 138 101 107 18156411 10.1093/jn/138.1.101

[B61] Voedingscentrum Richtlijnen Voedselkeuze (Nutrition Guidelines) 2011 Den Haag: Voedingscentrum

[B62] Ogden J Reynolds R Smith A Expanding the concept of parental control: a role for overt and covert control in children’s snacking behaviour? Appetite 2006 47 100 106 10.1016/j.appet.2006.03.330 16682098

[B63] De Coen V Vansteelandt S Maes L Huybrechts I De Bourdeaudhuij I Vereecken C Parental socioeconomic status and soft drink consumption of the child. The mediating proportion of parenting practices Appetite 2012 59 76 80 10.1016/j.appet.2012.03.024 22475631

[B64] Labree LJW van de Mheen H Rutten FFH Foets M Differences in overweight and obesity among children from migrant and native origin: a systematic review of the European literature Obes Rev 2011 12 e535 e547 10.1111/j.1467-789X.2010.00839.x 21348926

[B65] Lynch J Kaplan G Berkman L, Kawachi I Socioeconomic position Social epidemiology 2000 New York: Oxford University Press 13 35

[B66] Berkman L Kawachi I Social Epidemiology 2000 New York: Oxford University Press

[B67] Stevens J Applied Multivariate Statistics for the Social Sciences 1992 Hillsdale, NJ: Erlbaum

[B68] Gubbels JS Kremers SPJ Goldbohm RA Stafleu A Thijs C Energy balance-related behavioural patterns in 5-year-old children and the longitudinal association with weight status development in early childhood Public Health Nutr 2011 1 1 9 10.1017/S1368980011003089 22124196

[B69] Nunnally J Psychometric theory 1978 2 New York: McGraw-Hill

[B70] Baranowski T Smith M Baranowski J Wang DT Doyle C Lin LS Hearn MD Resnicow K Low validity of a seven-item fruit and vegetable food frequency questionnaire among third-grade students J Am Diet Ass 1997 97 66 68 10.1016/S0002-8223(97)00022-9 8990421

[B71] Van Assema P Brug J Ronda G Steenhuis I Oenema A A Short Dutch Questionnaire to Measure Fruit and Vegetable Intake: Relative Validity Among Adults and Adolescents Nutr Health 2002 16 85 106 10.1177/026010600201600203 12102370

[B72] Wendel-Vos G Reproducibility and relative validity of the short questionnaire to assess health-enhancing physical activity J Clin Epidem 2003 56 1163 1169 10.1016/S0895-4356(03)00220-8 14680666

